# Therapeutic efficacy of artemether–lumefantrine and artesunate–amodiaquine for the treatment of uncomplicated *Plasmodium falciparum* malaria in Mali, 2015*–*2016

**DOI:** 10.1186/s12936-021-03760-9

**Published:** 2021-05-25

**Authors:** Youssouf Diarra, Oumar Koné, Lansana Sangaré, Lassina Doumbia, Dade Bouye Ben Haidara, Mouctar Diallo, Ababacar Maiga, Hamadoun A. Sango, Halidou Sidibé, Jules Mihigo, Douglas Nace, Dragan Ljolje, Eldin Talundzic, Venkatachalam Udhayakumar, Erin Eckert, Celia J. Woodfill, Leah F. Moriarty, Pharath Lim, Donald J. Krogstad, Eric S. Halsey, Naomi W. Lucchi, Ousmane A. Koita

**Affiliations:** 1 University of Sciences, Techniques and Technologies of Bamako, Bamako, Mali; 2 Referral Health Centre of Sélingué, Ministry of Health and Public Hygiene, Bamako, Mali; 3 National Malaria Control Programme, Ministry of Health and Public Hygiene, Bamako, Mali; 4 U.S. President’s Malaria Initiative, USAID Office, Bamako, Mali; 5 Malaria Branch, Centers for Disease Control and Prevention, Atlanta, GA USA; 6 RTI International, Washington, DC USA; 7 U.S. President’s Malaria Initiative, Atlanta, GA USA; 8 Medical Care Development International, Silver Spring, MD USA; 9 Tulane School of Public Health and Tropical Medicine, New Orleans, LA USA

**Keywords:** Malaria, Antimalarial resistance, Efficacy, Mali, Artemether–lumefantrine, Artesunate–amodiaquine, *Pfk13*, *Pfcrt*, *Pfmdr1*

## Abstract

**Background:**

The current first-line treatments for uncomplicated malaria recommended by the National Malaria Control Programme in Mali are artemether–lumefantrine (AL) and artesunate–amodiaquine (ASAQ). From 2015 to 2016, an in vivo study was carried out to assess the clinical and parasitological responses to AL and ASAQ in Sélingué, Mali.

**Methods:**

Children between 6 and 59 months of age with uncomplicated *Plasmodium falciparum* infection and 2000–200,000 asexual parasites/μL of blood were enrolled, randomly assigned to either AL or ASAQ, and followed up for 42 days. Uncorrected and PCR-corrected efficacy results at days 28 and 42. were calculated. Known markers of resistance in the *Pfk13, Pfmdr1,* and *Pfcrt* genes were assessed using Sanger sequencing.

**Results:**

A total of 449 patients were enrolled: 225 in the AL group and 224 in the ASAQ group. Uncorrected efficacy at day 28 was 83.4% (95% CI 78.5–88.4%) in the AL arm and 93.1% (95% CI 89.7–96.5%) in the ASAQ arm. The per protocol PCR-corrected efficacy at day 28 was 91.0% (86.0–95.9%) in the AL arm and 97.1% (93.6–100%) in the ASAQ arm. ASAQ was significantly (p < 0.05) better than AL for each of the aforementioned efficacy outcomes. No mutations associated with artemisinin resistance were identified in the *Pfk13* gene. Overall, for *Pfmdr1*, the N86 allele and the N**F**D haplotype were the most common. The N**F**D haplotype was significantly more prevalent in the post-treatment than in the pre-treatment isolates in the AL arm (p < 0.01) but not in the ASAQ arm. For *Pfcrt*, the CV**IET** haplotype was the most common.

**Conclusions:**

The findings indicate that both AL and ASAQ remain effective for the treatment of uncomplicated malaria in Sélingué, Mali.

**Supplementary Information:**

The online version contains supplementary material available at 10.1186/s12936-021-03760-9.

## Background

Integrated strategies to control malaria have led to a decrease in the number of malaria cases and deaths. The incidence rate of malaria declined globally between 2010 and 2018, from 71 to 57 cases per 1000 population at risk though the statistical significance of this decline has not been reported [1]. Tracking efficacy and monitoring for parasite resistance helps ensure that anti-malarials, critical tools in malaria control, remain effective. In Mali, malaria is a major public health problem with a parasite prevalence varying from 38.3% among children with age between 6 and 8 months to 58% in older children between 48 and 59 months of age [2]. Seasonality is marked with a peak during the rainy season, which has led to the implementation of the seasonal malaria chemoprevention in Mali.

In 2001, treatment failure using chloroquine (CQ) was linked to an increase in child mortality in Africa [3]. In 2006, Mali stopped using CQ as first-line treatment for uncomplicated malaria. Consequently, artemisinin-based combination therapy (ACT) was adopted as per the World Health Organization (WHO) recommendation for the treatment of uncomplicated *Plasmodium falciparum* malaria [4]. ACT is designed so that the artemisinin derivative, with a relatively short half- life, acts rapidly to reduce the parasite burden, while the partner drug, having a longer half-life, continues to clear the parasites for days or weeks after treatment [4]. Therefore, during an efficacy study, it is important to monitor: (1) the clearance of parasites shortly after ACT administration, as slow clearance could indicate a possible failure of the artemisinin derivative and (2) recrudescent infection days or weeks after treatment, which may be associated with failure of the partner drug. The current Mali national malaria case management guidelines recommend the use of either artemether-lumefantrine (AL) or artesunate-amodiaquine (ASAQ) as first-line treatment for uncomplicated malaria. Because amodiaquine is reserved for seasonal malaria chemoprevention, used in combination with sulfadoxine–pyrimethamine, ASAQ is not used as a malaria treatment even though it appears as a first-line treatment for uncomplicated malaria in the country’s malaria treatment guidelines.

Declining efficacy of ACT in Southeast Asia (e.g., Cambodia, Thailand) has led to heightened concern for the spread of resistant parasites to other malaria endemic countries [5, 6] and the independent emergence of resistant mutants in other malaria endemic countries was observed in Guyana [7] and recently reported in Rwanda [8]. Although artemisinin resistance has not yet been reported in Africa [1], the intensive use of ACT may drive a strong selective pressure for artemisinin resistance. Thus, it is necessary to routinely monitor the therapeutic efficacy of ACT.

A handful of studies over the past decade have investigated the efficacy of AL in Mali and reported PCR-corrected efficacies greater than 98%. These include: a 2010–2014 study in Sotuba, Kollé, and Banambani [9]; a 2012–2014 study in Dioro [10]; and a 2013–2015 study in Donéguébougou and Torodo [11]. Although two of these studies [9, 11] considered reinfections as treatment successes in their calculation of PCR-corrected efficacy (an approach not consistent with WHO guidelines), the efficacy results remained above 97% when recalculating efficacy using the WHO-recommended approach. Efficacy results above 90% were reported in another Mali study [12], conducted in 2013–2015, although this study reported combined results in children from another country, Niger, in addition to Koulikoro, Mali. The most recent publication on the efficacy of ASAQ in Mali is from a study conducted 2005–2007 in Bougoula-Hameau [13]. This study reported a 98.7% day 28 PCR-corrected efficacy (the rate was 98.3% when recalculated per WHO methodology) and a 78.5% day 28 uncorrected efficacy. Other artemisinin-based combinations endorsed by WHO for treatment of uncomplicated malaria have been shown to have PCR-corrected efficacies greater than 90% in Mali, including dihydroartemisinin–piperaquine (DP) [11], artesunate–pyronaridine [14], and artesunate plus sulfadoxine–pyrimethamine [9].

Surveillance of molecular markers associated with the resistance of *P. falciparum* parasites to anti-malarial drugs provides an additional way of assessing whether efficacy may be threatened. For instance, certain mutations in the propeller domain of the *P. falciparum kelch 13* (*Pfk13)* gene are associated with artemisinin partial resistance [15], in particular 10 single nucleotide polymorphisms (SNPs) are validated as markers of partial resistance to artemisinin [16]: F446**I**, N458**Y**, M476**I**, Y493**H**, R539**T**, I543**T**, P553**L**, R561**H**, P574**L**,and C580**Y**. To date, there have been no reports of *Pfk13* polymorphisms associated with artemisinin partial resistance in Mali. Polymorphisms in *P. falciparum* multidrug resistance-1 (*Pfmdr-1)* and *P. falciparum* chloroquine resistance transporter (*Pfcrt)* genes are associated with recurrent parasitaemia after treatment with AL and ASAQ [17]. In Africa, the most relevant polymorphisms of *Pfmdr1* are found at codons N86**Y**, Y184**F**, and D1246**Y** [17]. The 86**Y** mutation has been associated with decreased chloroquine and amodiaquine sensitivity while the N86 has been implicated in decreased sensitivity to lumefantrine. The N86, 184**F**, and D1246 (N**F**D) haplotype is associated with decreased sensitivity to AL, while the 86Y, Y184, and 1246Y (**Y**Y**Y**) haplotype is reported to be associated with decreased sensitivity to ASAQ [17]. The most commonly reported *pfcrt* mutations are observed in codons 72, 74–76 [18]. Parasites with the wild type CVMNK haplotype are sensitive to CQ treatment, while the CV**IET** haplotypes are associated with chloroquine resistance. The *Pfcr*t 76 T mutation has also been shown to play a role in amodiaquine resistance in some studies in Africa [17]. Prior studies have demonstrated the selection of this allele after amodiaquine monotherapy [19] or ASAQ treatment [17, 20–22].

To fulfill the WHO recommendation of performing biannual monitoring of parasite susceptibility to anti-malarial drugs in *P. falciparum* endemic areas, a therapeutic efficacy study of AL and ASAQ for the treatment of uncomplicated malaria was carried out. In addition, the study investigated the prevalence of molecular markers associated with resistance and reduced susceptibility to AL and ASAQ.

## Methods

### Study sites

**Fig. 1:**
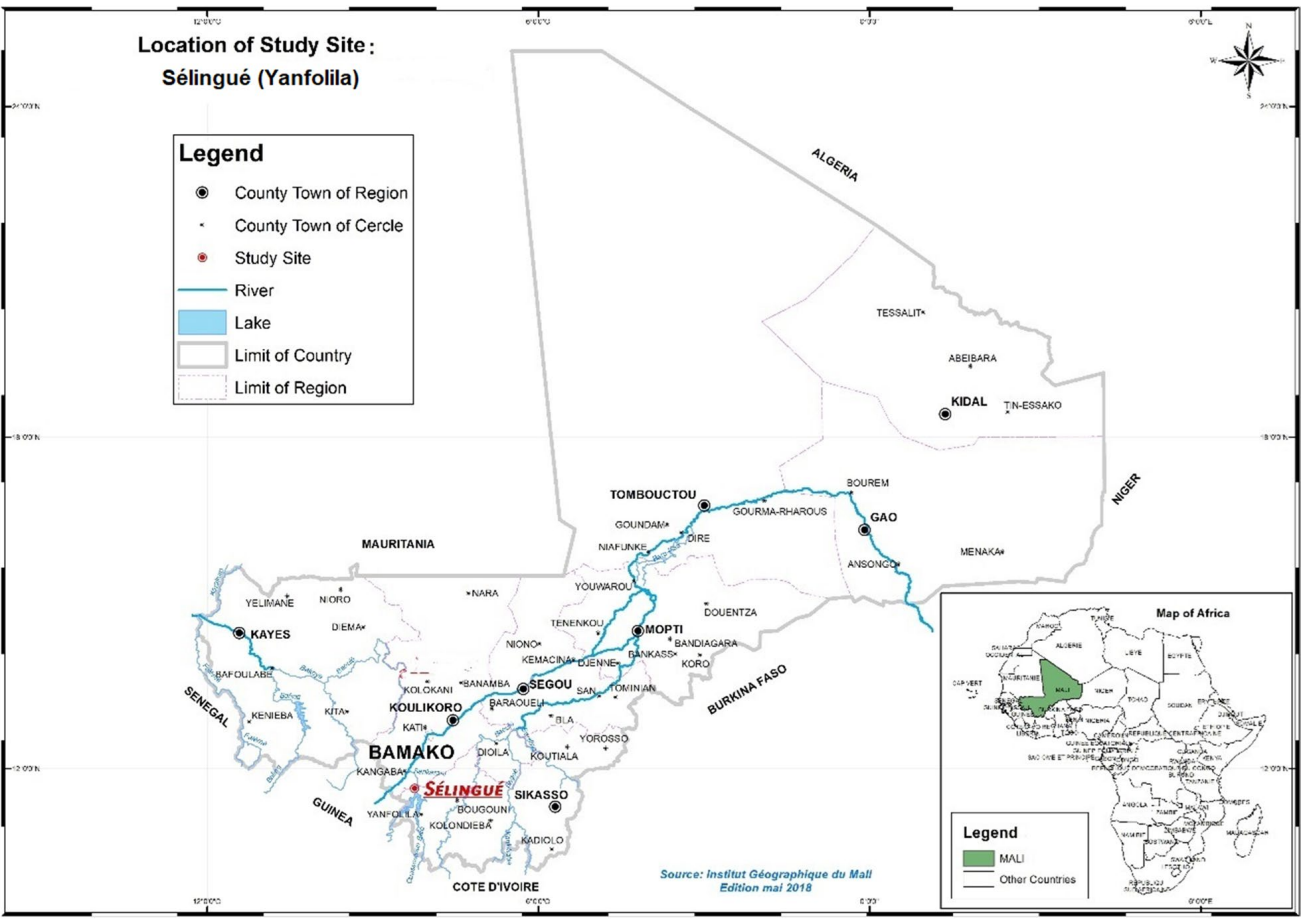
The Sélingué study site (in red font) used in the Mali therapeutic efficacy study, 2015–2016 (Institut National Geographique du Mali, May 2018)

The study was conducted in Sélingué, a commune in the southern part of Mali in the Sikasso region (140 km northwest of Bamako; Fig. 1), where malaria transmission is high due to a relatively long rainy season and the presence of a lake formed by a hydroelectric dam on the Sankarani River. In 2011, malaria parasite prevalence was 42.7% in children aged five to nine years in Sélingué [23]. Malaria incidence in this area is highest between September and January. The study team (two physicians, two biologists, and two research pharmacists) was stationed in the health centre during the entire implementation of the study.

### Study design and participants recruitment

The efficacy of AL and ASAQ was assessed in a prospective two-arm study conducted from November 2015 to November 2016, as per the WHO protocol for surveillance of anti-malarial drug efficacy [24]. Participants were recruited in the health district of Sélingué among children between 6 and 59 months of age with an axillary temperature ≥ 37.5 °C or a history of fever during the previous 24 h, haemoglobin > 5 g/dl, and uncomplicated *P. falciparum* infection with 2000–200,000 asexual parasites/μL of blood based on microscopy. Children with severe malaria, severe anaemia (haemoglobin ≤ 5 g/dL), weight less than 9.0 kg, the presence of any acute comorbidity (pneumonia and malnutrition), or with a history of taking anti-malarial drugs in the four past days were not included in the study and were referred to the health centre for management. In addition, those children whose parents refused to participate or lived outside the radius of 5 km from Sélingué Health centre were excluded from the study. After consent was obtained from parents, enrolled patients were randomly assigned to either the AL or ASAQ arm, with a ratio of 1:1, using a random number generator with PASS11 (pass power analysis and sample size software, NCSS statistical software, https://www.ncss.com/). The study was single blinded and the randomization was implemented by the research pharmacist. Dosage was weight-based according to manufacturer recommendations. Clinic staff, including physicians and laboratory personnel involved in the study, were blinded to the allocation except for the research pharmacist who provided the treatment. The research pharmacist administered all treatment drugs to each patient on days 0 (day of enrollment), 1, and 2. Blood samples were collected on the day of enrollment (pre-treatment); days 1, 2, and 3; and weekly from days 7 to 42. Participants were followed up with physical and clinical exams after anti-malarial administration to evaluate for adverse medication effects or recurrent parasitaemia. Enrolled patients were requested to return to the clinic if they experienced any symptoms of malaria during the follow-up period. The study schedule was available to all parents of each study participant. A blood slide for microscopy and dried blood spots on filter paper were prepared any time a blood sample was collected.

### Microscopy

Thick and thin malaria blood smears were prepared on glass slides and stained with 3% Giemsa (Sigma; St. Louis, MO) in phosphate buffer (pH 7.0). The slide reading was performed independently by two experienced microscopists at the study sites. Parasite density was calculated according to well-established methods [25], briefly, asexual parasite densities was estimated by counting parasites against 200 white cells, assuming a standard leukocyte count of 8000/µl. If a discrepancy in the parasite density between these two readers exceeded 20% or differed by *Plasmodium* species, a third slide reading was performed by another senior microscopist and the closest readings averaged for a final parasite count.

### Sample size estimation

A sample size of 240 patients per arm was targeted in order to provide adequate power to estimate an efficacy of 95% with a confidence interval of ± 5%, assuming an expected efficacy of 95% and a maximum loss to follow-up and withdrawal rate of 20%. This was based on the ability of the drug to clear parasitaemia at day 7 or before and give enough sample size to compare the efficacy of the two treatment arms.

### Clinical monitoring

Each enrolled child received a three-day course of either AL (Coartem, Novartis) or ASAQ (ASAQ-Denk, fixed dose, DENK PHARMA, Munchen, Germany). All doses administered were given under the direct observation of the study pharmacist at the Sélingué health centre and both AL and ASAQ were given with a peanut-based paste and milk. For ASAQ-Denk fixed dose, the dosage was adjusted based on the patient’s body weight (as recommended in the package insert), 2 to 10 mg of artesunate per kg; 7.5 to 15 mg of amodiaquine once daily for 3 days. Enrolled participants randomly assigned to AL (Coartem®, Novartis), received a weight-adjusted dosing of 20 mg of artemether and 120 mg of oral lumefantrine twice a day for 3 days as recommended in the insert package, 1 tablet for 5–14 kg, 2 tablets for 15–24 kg, and 3 tablets for 25–34 kg. All children were observed for a minimum of one hour after each dose to monitor for vomiting or other side effects. In case of vomiting, another dose of the drug was then administered. After the three days of dosing, each enrolled child was followed on days 7, 14, 21, 28, 35, and 42. During each visit, the following were performed or collected: physical exam (weight, height), temperature, thin and thick blood smears, and dried blood spots. Haemoglobin levels were measured at the time of enrollment and on day 42, at the completion of the study. All blood specimens were collected by finger prick. In cases of recurrent parasitaemia, quinine was administered according to the guidelines of the Mali National Malaria Control Programme.

### Study outcome classification

Treatment responses were classified following the WHO guidelines [24]. Treatment outcomes were classified as either early treatment failure (ETF: day 2 count higher than pre-treatment; or day 3 count ≥ 25% of count on pre-treatment), late clinical failure (LCF: danger signs or severe malaria in the presence of parasitaemia on any day between day 4 and day 42 with axillary temperature ≥ 37.5 °C in patients who did not previously meet any of the criteria of ETF), late parasitological failure (LPF: presence of parasitaemia on day between day 7 and day 42 with axillary temperature ≤ 37.5 °C in patients who did not previously meet any of the criteria of ETF or LCF), or adequate clinical and parasitological response (ACPR: absence of parasitaemia on day 28 or 42, irrespective of axillary temperature in patients who did not previously meet any of the criteria of ETF, LCF, or LPF) as per WHO definitions [3], before and after PCR correction. Children experiencing ETF without danger signs or severe malaria were not offered retreatment. However, children meeting criteria for ETF were not included in the numerator (i.e., as treatment successes) when calculating ACPR.

### PCR-correction

Genotyping using the merozoite surface proteins 1 and 2 (*msp1* and *msp2),* and glutamine-rich protein (*glurp)* markers, and Sanger sequencing of *P. falciparum* parasites was performed on samples obtained from participants on enrollment day (pre-treatment) and on the day of recurrent parasitaemia (post-treatment). Genomic DNA was extracted from all collected samples using the QiAamp mini kit (Qiagen, Valencia, CA USA) following the manufacturer’s instructions. Primers designed to amplify three allelic families from block 2 of *msp1* (K1, MAD20 and RO33), two allelic families from *msp2* (FC27 and IC/3D7), and the polymorphic region of *glurp* were used in PCR amplification and analysis as previously described [26, 27]. Band sizes were scored using an automated Gel Image system (UVP, Upland, CA, USA) and compared across the three markers for paired pre-treatment and day of recurrence samples. For *msp1* and *msp2*, bands with lengths within 10 base pairs were considered a match; for *glurp*, lengths within 50 base pairs were considered a match. If there was at least one matching band in any allelic family for all three markers, the recurrence was classified as a recrudescence (regardless of whether there were additional or missing alleles). If there were no shared alleles for at least one marker, the recurrence was classified as a reinfection. If there were no amplification products resulting in sharp, defined bands in both the pre-treatment and day of recurrence samples for a gene, that gene was not used to distinguish between recrudescence and reinfection, but the aforementioned classification criteria were applied for the genes that were amplified.

### Molecular markers of drug resistant

Paired pre-treatment and day of recurrent parasitaemia samples were assessed for known markers of resistance in the *Pfcrt*, *Pfk13,* and *Pfmdr1* genes. In addition, all pre-treatment samples were assessed for mutations in the *Pfk13* and *Pfmdr1* genes. Fragments of *Pfk13*, *Pfcrt,* and *Pfmdr1* were amplified by nested PCR using previously published primers [28, 29]. Direct Sanger sequencing of the nested purified PCR products was performed by using a BigDye Terminator v3.1 cycle sequencing kit on an iCycler thermal cycler (Bio-Rad, California, USA). Sequence analysis was performed using Geneouis R7 (Biomatters, Auckland, New Zealand). The *Pfcrt* codons 72, 74, 75, and 76, *Pfk13* propeller domain (codon positions: 389–649), and *Pfmdr1* codons 86, 184, and 1246 were analysed for SNPs. The *P. falciparum* laboratory strain 3D7 *Pfcrt, Pfk13,* and *Pfmdr1* were used as reference sequences for the analysis. Molecular analyses were performed in collaboration with the U.S. Centers for Disease Control and Prevention (CDC) Malaria Laboratory in Atlanta, USA, as part of the President’s Malaria Initiative (PMI)-supported Anti-malarial Resistance Monitoring in Africa Network [30].

### Statistical analysis

Both per protocol and Kaplan–Meier analyses were conducted to evaluate the study outcome data. The main difference between the two is the Kaplan–Meier approach takes into account patients who were withdrawn from the study, such as for acquiring a reinfection, and censors them at the day of reinfection, whereas the per protocol approach removes those with reinfection from the analysis altogether in the PCR-corrected calculations. Treatment responses were classified following the WHO guidelines [24]. Uncorrected efficacy rates were calculated by dividing the number of participants with an ACPR in each arm by the total number of participants. For the PCR-corrected efficacy, per the WHO protocol, new infections identified during follow-up were not considered as treatment successes or failures and were excluded from the corrected estimations of treatment efficacy [24]. Therefore, patients were censored or excluded from the PCR-corrected analyses if the PCR results indicated that the failure was due to reinfection with *P. falciparum*. Uncorrected and PCR-corrected ACPR for AL and ASAQ were compared at both day 28 and 42 using a chi-square test. The capacity of the two ACTs to clear parasites by day 7 and the post-treatment prophylactic effect of the two treatments was evaluated using a previously described approach by Koita et al*.* [31]. This approach involved comparing clinical cure rates, a composite of clearance of asexual parasites and fever by day 7. The post-treatment prophylactic effects of the ACT was assessed by including new infections in the denominator of the uncorrected efficacy estimations. Kaplan Meier estimates were calculated for the uncorrected efficacy, the PCR-corrected efficacy for risk of recrudescence (per WHO guidelines [24]), and the PCR-corrected efficacy for reinfections only (using an approach described in [31]). Corresponding survival curves and hazard ratios comparing AL and ASAQ were also generated.

Point mutations in the *Pfk13, Pfmdr1,* and *Pfcrt* genes were reported as single or mixed (wild-type and mutant) infections. For samples with mixed infections and SNP variations at multiple sites, each possible haplotype constructed from the observed SNPs was reported for *Pfmdr1*. In reporting *Pfcrt* haplotypes in samples with a mixed infection and SNP variations at multiple sites, the wild-type (CVMNK) and most likely mutant type (e.g., CV**IET**) were reported. The prevalence of the *Pfmdr1* mutant alleles and haplotypes was calculated stratifying by the treatment arms (AL and ASAQ) and compared between the pre-treatment samples (samples with ACPR and samples from subjects that later had reinfections) and post-treatment samples (reinfections and recrudescent infections). Likewise, the prevalence of the *Pfcrt* mutant alleles and haplotypes was compared between pre-treatment and post-treatment samples in the ASAQ arm; only samples from the ASAQ arm were used because of the known association between *Pfcrt* and amodiaquine efficacy [17]. For these analyses, pre-treatment samples from participants with recrudescent infections were excluded. Differences between pre-treatment and post-treatment samples were assessed using Fisher’s Exact test. The prevalence of wild type *versus* mutant alleles were compared between groups (e.g., *Pfmdr1* N86 versus 86Y). Mixed infections were excluded. To compare haplotypes, the sum of samples with the predominant haplotype was compared to the sum of those without that haplotype (e.g., *Pfmdr1* haplotype NFD *versus* all other haplotypes). Statistical significance was defined as p < 0.05 for all statistical tests. Analyses were performed using Graph Pad Prism version 6.00 for Windows (Graph Pad Software, La Jolla, California, USA) and R (R Foundation for Statistical Computing, Vienna, Austria).

### Ethical considerations

Ethical review and approval was obtained from the Internal Review Board of the Institut National de Recherche en Santé Publique (INRSP, Ministry of Health and Public Hygiene, FWA 00000892). Work performed at the Centers for Disease Control and Prevention was deemed to not constitute engagement in human subject research (CDC, Atlanta, USA; CGH tracking #2016-012). Parents or guardians of study participants were asked to provide written informed consent.

## Results

### Baseline characteristics of study participants

**Fig. 2:**
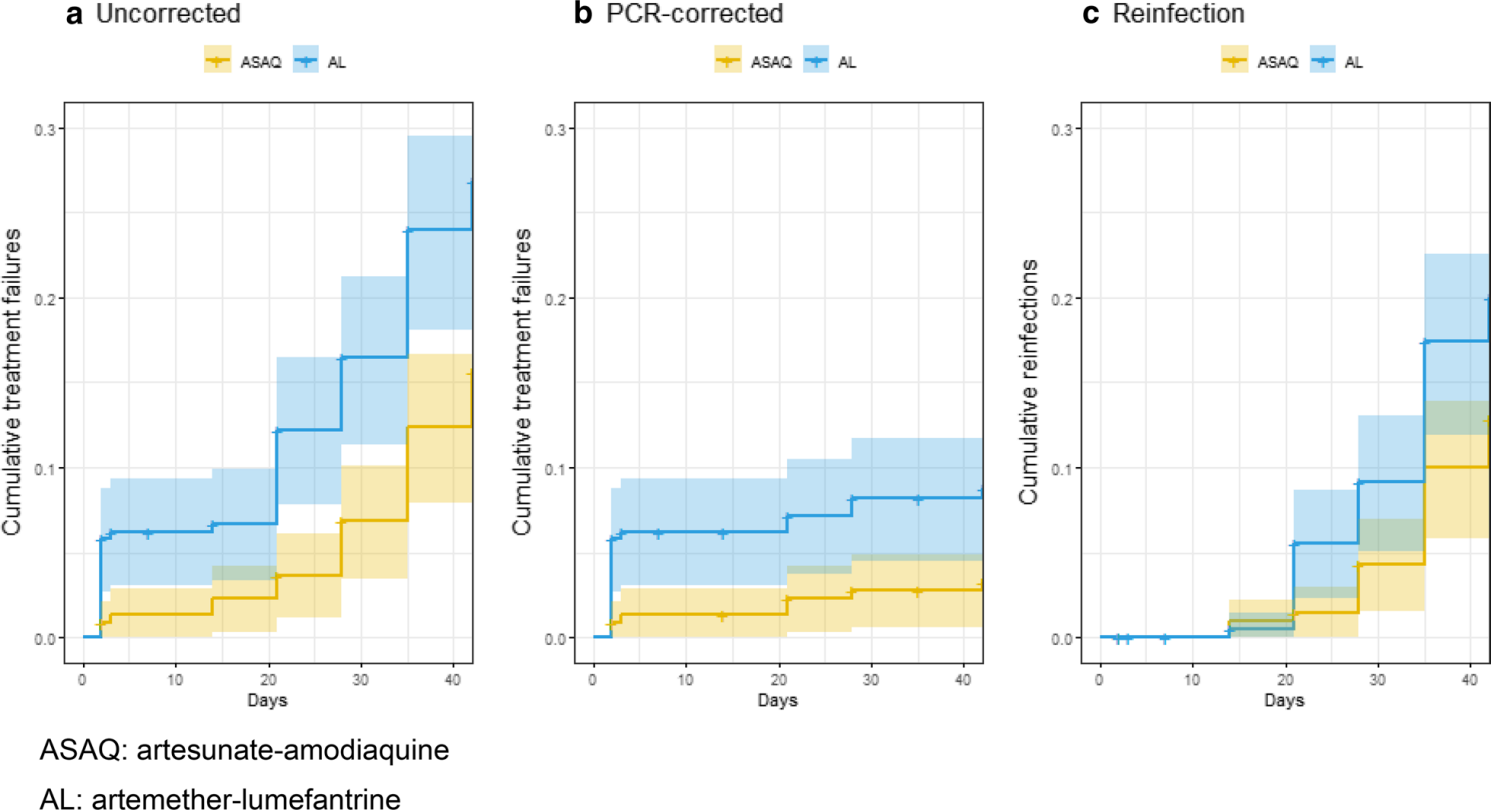
Kaplan Meier graphs of rates of **a** uncorrected treatment failures, **b** recrudescent infections (PCR-corrected), and c) reinfections during 42 days of therapeutic efficacy monitoring for two ACTs in Sélingué, Mali, 2015–2016

A total of 3732 patients were screened for the study and, of these, 449 were enrolled, 225 in the AL group and 224 in the ASAQ group (Fig. 2). A majority of the screened patients did not meet the study criteria for inclusion. Common reasons for exclusion were high parasitaemia (greater than 200,000 parasites/µl), low haemoglobin (≤ 5 g/dl), low weight (< 9 kg), and not having malaria on screening microscopy. For parameters such as age, weight, gender, parasitaemia, and haemoglobin at the time of enrollment, the two groups were comparable (Table 1; p > 0.05). Loss to follow-up was comparable (p > 0.05) between the two groups at days 28 and 42. The two medications were well-tolerated, and no children experienced any serious adverse effects from either AL or ASAQ.

**Table 1:** Participants characteristics at enrollment and follow-up, Mali 2015–2016

Study arm	AL (225)	ASAQ (224)	p-value
Participant characteristics at baseline			
Median age, month (range)	36.0 (7–59)	36.0 (6–59)	0.970
Median weight, kg (range)	13.0 (9–21)	13.3 (9–20)	0.872
Number male/female	126/99	111/113	0.083
Median pre-treatment parasitemia, parasites/µL (range)	11,750 (2000–199,800)	11,825 (2000–197,500)	0.659
Median pre-treatment hemoglobin (g/dL)	9.4 (5–13)	9.0 (5–13)	0.161

AL: artemether–lumefantrine; ASAQ: artesunate–amodiaquine

### Efficacy outcomes

The ETF rate was 6.5% in the AL group; this was higher than the rate in the ASAQ group (1.4% [p < 0.01]). Both AL and ASAQ were able to clear parasitaemia before or on day 7 with 99.5% and 100% clearance rates, respectively (post-treatment prophylaxis). A total of 75 patients had recurrent infections during the 42-day follow-up period, 34 of whom had recurrent infections during the first 28 days. The uncorrected efficacy rates on days 28 and 42 were higher in the ASAQ group than in the AL group (Table 2).

**Table 2:** Uncorrected and PCR-corrected treatment outcomes at days 28 and 42 for artemether-lumefantrine (AL) and artesunate-amodiaquine (ASAQ), Mali 2015–2016

	AL		95% CI		ASAQ		95% CI			p-value^†^
			Lower	Upper			Lower		Upper	
Uncorrected treatment outcomes—day 28										
Number reaching an endpoint	217				217					
Early treatment failure	14	6.5%	3.2%	9.7%	3	1.4%	0.0%	2.9%		0.006
Late clinical failure	10	4.6%			5	2.3%				
Late parasitological failure	12	5.5%			7	3.2%				
Adequate clinical and parasitological response at day 28	181	83.4%	78.5%	88.4%	202	93.1%	89.7%	96.5%		0.002
Uncorrected treatment outcomes—day 42										
Number reaching an endpoint	212				214					
Early treatment failure	14	6.6%	3.3%	9.9%	3	1.4%	0.0%	3.0%		0.006
Late clinical failure	15	7.1%			9	4.2%				
Late parasitological failure	29	13.7%			22	10.3%				
Adequate clinical and parasitological response at day 42	154	72.6%	66.6%	78.6%	180	84.1%	78.3%	89.9%		0.004
PCR corrected treatment outcomes—day 28										
Number reaching an endpoint	217				217					
Early treatment failure	14	6.5%	3.2%	9.7%	3	1.4%	0.0%	2.9%		0.006
Recrudescent cases	4	1.8%			3	1.4%				
Total new infections	18	8.3%			9					
Day 7–14	1				2					
Day 15–21	10				1					
Day 22–28	7				6					
Adequate clinical and parasitological response at day 28	181	91.0%	86.0%	95.9%	202	97.1%	93.6%	100.0%		0.008
PCR corrected treatment outcomes—day 42										
Number reaching an endpoint	212				214					
Early treatment failure	14	6.6%	3.3%	9.9%	3	1.4%	0.0%	3.0%		0.006
Recrudescent cases	5	2.4%			4	1.9%				
Total new infections	39	18.4%			27	12.6%				
Day 7–14	1				2					
Day 15–21	10				1					
Day 22–28	7				6					
Day 29–35	16				12					
Day 36–42	5				6					
Adequate clinical and parasitological response at day 42	154	89.0%	83.0%	95.0%	180	96.3%	91.0%	100.0%		0.008

^†^p < 0.05

The day 28 uncorrected efficacy was 83.4% (95% CI 78.5–88.4%) for AL and 93.1% (95% CI 89.7–96.5%) for ASAQ, which was significantly different (p < 0.01; Table 2), a finding also significant at day 42 (p < 0.01). The per-protocol PCR-corrected efficacy rates at day 28 was 91.0% (95% CI 86.0–95.9%) for AL and 97.1% (95% CI 93.6–100%) for ASAQ, which was significantly different between the two groups (p < 0.01), a finding also significant at day 42 (p < 0.01). The majority of recurrent parasitaemias during the 42-day follow-up period were classified as new infections rather than recrudescent infections, 88.6% (39/44) for AL and 87.1% (27/31) for ASAQ, most of which occurred after 28 days.

**Fig. 3:**
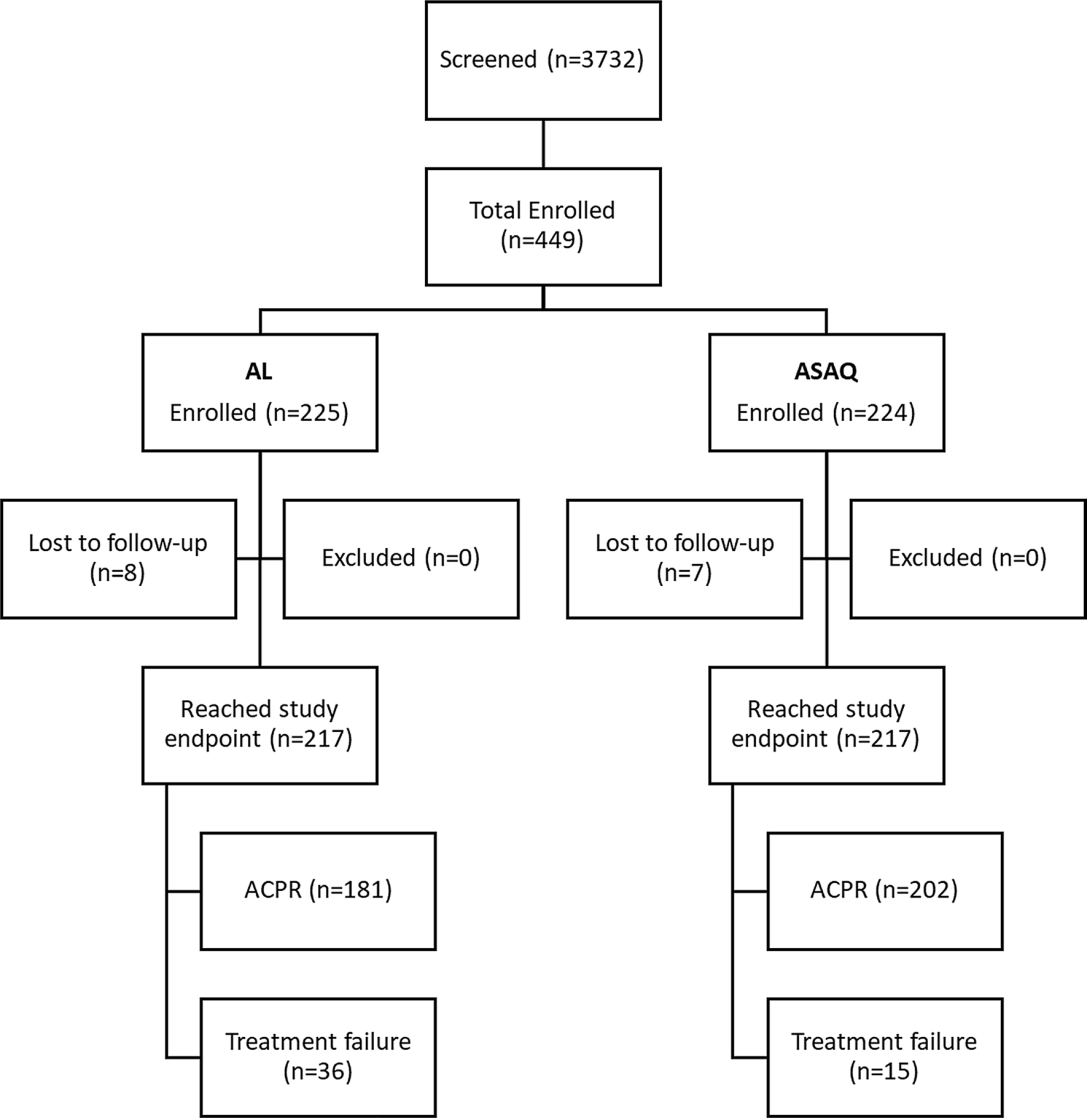
Patient screening and enrollment

The uncorrected day 28 Kaplan Meier estimates were 83.6% (95% CI 78.8–88.6%; Additional file 1: Table S1) for AL and 93.2% (95% CI 89.9–96.6%) for ASAQ. The PCR-corrected day 28 Kaplan Meier estimates were 91.9% (95% CI 88.3–95.5%) for AL and 97.3% (95% CI 95.1–99.4%) for ASAQ. The day 28 cumulative risk of treatment failure was statistically significantly higher for the AL arm than the ASAQ arm for risk of reinfections plus recrudescences (uncorrected; hazard ratio 2.58), recrudescences only (PCR-corrected; hazard ratio 3.12), and reinfections only (hazard ratio 2.59; Fig. 3 and Additional file 1: Table S1). Results of genotyping using the *msp1*, *msp2,* and *glurp* genes can be found in Additional file 1: Table S2.

**Table 3:** Prevalence of *Pfmdr1* polymorphisms in pre-treatment and post-treatment samples stratified by treatment arms, therapeutic efficacy monitoring, Mali, 2015–2016

	AL arm			ASAQ arm		
	Pre-treatment n, (%)	Post-treatment n, (%)	p-value*	Pre-treatment n, (%)	Post-treatment n, (%)	p-value*
*Pfmdr1* point mutations						
*Pfmdr1 codon 86*	n = 126	n = 36		n = 120	n = 27	
N86	103 (81.7)	35 (97.2)	0.1954	97 (80.8)	22 (81.5)	0.767
86N/**Y**	9 (7.1)	0 (0.0)		5 (4.2)	2 (7.4)	
86**Y**	14 (11.1)	1 (2.8)		18 (15.0)	3 (11.1)	
*Pfmdr1* codon 184	n = 144	n = 36		n = 140	n = 26	
Y184	44 (30.6)	7 (19.4)	0.1029	38 (27.1)	5 (19.2)	0.464
184Y/**F**	19 (13.2)	0 (0.0)		15 (10.7)	3 (11.5)	
184**F**	81 (56.3)	29 (80.6)		87 (62.1)	18 (69.2)	
*Pfmdr1* codon 1246	n = 152	n = 39		n = 146	n = 25	
D1246	149 (98.0)	39 (100.0)	1	144 (98.6)	24 (96.0)	1
1246D/**Y**	0 (0.0)	0 (0.0)		0 (0.0)	1 (4.0)	
1246**Y**	3 (2.0)	0 (0.0)		2 (1.4)	0 (0.0)	
*Pfmdr1* haplotypes^†^	n = 124	n = 35		n = 115	n = 24	
N**F**D	74 (59.7)	27 (77.1)*	0.014	71 (61.7)	17 (70.8)	1
NYD	56 (45.2)	7 (20.0)		39 (33.9)	6 (25.0)	
** YF**D	20 (16.1)	1 (2.9)		19 (16.5)	4 (16.7)	
N**FY**	0 (0.0)	0 (0.0)		0 (0.0)	1 (4.2)	
NY**Y**	0 (0.0)	0 (0.0)		0 (0.0)	1 (4.2)	
** Y**YD	4 (3.2)	0 (0.0)		2 (1.7)	1 (4.2)	
**YFY**	1 (0.8)	0 (0.0)		0 (0.0)	1 (4.2)	
** Y**Y**Y**	2 (1.6)	0 (0.0)		2 (1.7)	1 (4.2)	

Bold letter denotes an encoded amino acid change

^†^Haplotypes defined at codons 86, 184, and 1246; haplotype numerators include isolates with mixed infections

^*^Comparing wild-type vs. mutant (excluding mixed infections) for point mutations and NFD versus sum of non-NFD for haplotypes

### Molecular markers of drug resistance

A total of 296/386 (76.6%) samples were successfully sequenced (23.4% of the samples did not give interpretable data likely due to low parasite density) for polymorphisms in the *Pfk13* gene (248 pre-treatment and 48 post-treatment samples). Any of the known mutations associated with artemisinin partial resistance in the *Pfk13* gene was not observed. However, we observed synonymous polymorphisms in some pretreatment samples at codons: T478**T**, Y493**Y**, K503**K**, and Q613**Q**, all in one sample each. The synonymous polymorphism C469**C** was found in two samples and the A578**S** mutant allele was observed in two samples (pre-treatment and day 28) collected from the same patient.

A total of 388 samples were sequenced for the *pfmdr1* gene (314 pre-treatment and 74 post-treatment samples). Overall, the wildtype N86 allele was found in a majority of the successfully sequenced pre-treatment (200/246, 81.3%) and post-treatment (57/63, 90.5%) isolates. Similarly, the overall prevalence of the 184**F** allele was high in pre-treatment (168/284, 59.2%) and in the post-treatment isolates (47/62, 75.8%; Table 3). The N**F**D haplotype was overrepresented in the post-treatment compared to the pre-treatment isolates in the AL arm (77.1% vs 59.7%, p < 0.01) but not in the ASAQ arm (70.8% vs 61.7%, p = 1.00). A similar trend was observed for the prevalence of the N86 allele, which was higher in the post-treatment (97.2%) than the pre-treatment (81.7%) isolates in the AL arm, although the difference was not statistically different (p = 0.195). The prevalence of the N86 allele was similar in the pre-treatment (80.8%) and post-treatment (81.5%) isolates in the ASAQ arm (p = 0.766) (Table 3).

**Table 4:** Prevalence of *Pfcrt* polymorphisms in paired pre-treatment and post-treatment samples in the artesunate-amodiaquine treatment arm, therapeutic efficacy monitoring, Mali, 2015 – 2016

	Pre-treatment n, (%)* n = 26	Post-treatment n, (%) n = 30	p-value**
*Pfcrt* point mutations			
*Pfcrt* codon 72			
C72	26 (100.0)	30 (100.0)	NA
*Pfcrt* codon 73			
V73	26 (100.0)	30 (100.0)	NA
*Pfcrt* codon 74			
M74	4 (15.4)	16 (53.3)	0.018
74M**/I**	7 (26.9)	2 (6.7)	
74**I**	15 (57.7)	12 (40.0)	
*Pfcrt* codon 75			
N75	4 (15.4)	16 (53.3)	0.018
75N/**E**	7 (26.9)	2 (6.7)	
75**E**	15 (57.7)	12 (40.0)	
*Pfcrt* codon 76			
K76	4 (15.4)	16 (53.3)	0.018
76K/**T**	7 (26.9)	2 (6.7)	
76**T**	15 (57.7)	12 (40.0)	
*Pfcrt* haplotypes^†^			
CV**IET**	22 (84.6)	14 (46.7)	0.082
CVMNK	11 (42.3)	18 (60.0)	

Bold letter denotes an encoded amino acid change

^†^Haplotype totals include mixed infections

^*^4 recrudescent infections not included

^**^ Comparing wild-type vs. mutant (excluding mixed infections) for point mutations and CV**IET** versus CVMNK haplotypes

The *Pfcrt* gene was investigated in pre-treatment and post-treatment matched pairs of samples in the ASAQ arm of the study. A total of 60/62 (97%) samples were successfully sequenced. Mutations were observed in the M74**I**, N75**E** and K76**T** codons. The *Pfcrt* 76**T** allele, associated with amodiaquine resistance, was found in 57.7% of pre-treatment and 40.0% of post-treatment isolates, (p = 0.018; Table 4). Mixed infections with 76 K/**T** were found in 7 (26.9%) and 2 (6.7%) of pre-treatment and post-treatment isolates, respectively. All isolates sequenced harboured either the mutant CV**IET** or wild-type CVMNK haplotypes.

## Discussion

Even though this study investigated efficacy results through 42 days, WHO recommends reporting efficacy for AL and ASAQ at day 28, with 90% being the threshold where a change in first-line treatment should be considered [24]. The day 28 PCR-corrected efficacies for both AL (91.0%) and ASAQ (97.1%) were above this threshold. This is in concordance with previous studies conducted in Mali where the efficacy of AL was shown to be above 90% [33, 34]. However, when accounting for a 95% confidence interval, the 86.0–95.9% PCR-corrected efficacy range of AL indicates that continued frequent efficacy monitoring of this drug is warranted in Mali. These findings are not unlike those from Uganda [35], Angola [36], and Burkina Faso [37], which have also shown AL not performing as well as other artemisinin-based combinations, such as ASAQ and DP. Similar to the AL day 28 uncorrected efficacy of 83.4% in this study, two other studies from Mali examining day 28 uncorrected efficacy reported results of 83.8% and 84.5% [33, 34] and the study performed in Burkina Faso by Tinto & al, gave 58.4% for ASAQ and 46.1% for AL [32].

Although there were 14 and 3 ETFs in the AL and ASAQ arms, respectively, nearly all subjects cleared parasitaemia by day 7, implying that both treatments were able to clear parasites early in the infection. Compared with AL, the higher day 28 and 42 uncorrected efficacies of ASAQ may be a result of the longer half-life of amodiaquine compared with lumefantrine [4]. For both drug arms, a majority of reinfections occurred between days 29–42 compared with days 7–28. Artemisinin-based combinations containing a partner drug with a longer half-life, such as piperaquine in DP [4], may confer an even longer window of protection against a future infection. One limitation in this study was the dosing of ASAQ with fatty food, which the package insert recommends against; however, the efficacy of ASAQ was still adequate.

No *Pfk13* mutations associated with artemisinin resistance were found in any of the parasites investigated, consistent with other studies carried out in Africa where malaria parasites are largely wildtype in the *Pfk13* propeller domain and remain sensitive to artemisinin derivatives [15, 38]. However, other *Pfk13* polymorphisms were detected at six positions. The A578**S** polymorphism has been found throughout Africa and so far has not been associated with resistance to artemisinin derivatives but appears as a common polymorphism observed in African parasites [15, 38, 39]. A mutation at position 493 (Y493**H**) is one of the *PfK13* mutations associated with artemisinin resistance [15]*;* however, we found a silent mutation at this codon, Y493Y (TAC → TAT), in one sample. While it is possible this is a transitory mutation that could lead to the artemisinin resistant allele Y493**H**, no data currently exists to support this notion.

Equally important in the efficacy of an ACT is the partner drug. While delayed clearance (within 3 days post treatment) of parasites is associated with resistance to the artemisinin component, parasite recrudescence may occur when resistance to the partner drug exists, leading to an inadequate clinical and parasitological response. It is, therefore, imperative that resistance markers to partner drugs are evaluated. Point mutations in the *Pfcrt* and *Pfmdr1* genes are associated with decreased sensitivity to lumefantrine and amodiaquine [17]. In particular, the prevalence of the *Pfmdr1* N86 allele and N**F**D haplotype have been shown to be associated with reduced susceptibility to lumefantrine in some studies. In agreement with previous studies conducted in other parts of Africa [20, 29, 40], including Mali [41], a higher prevalence of the N**F**D haplotype in the post-treatment isolates in the AL arm was observed compared to pre-treatment isolates. This was not observed in the ASAQ arm, suggesting that parasites harbouring the N**F**D haplotypes are likely to be more tolerant to AL treatment, consistent with previous observations. Overall, the prevalence of the N86 allele was more than 80% in the pre-treatment isolates in our study, similar to what was recently reported in two study sites in Mali, Dangassa and Nioro-du-Sahel [41]. In addition, a majority (59.2%) of the baseline isolates in our study harboured the 184**F** allele, which can be contrasted with previous reports from Dangassa (39.9%) and Nioro-du-Sahel (48.2%). The significance of the high prevalence of N86 is unclear and may simply reflect the withdrawal of CQ drug pressure that selected for the 86**Y** polymorphism as previously indicated [42] and/or a true reflection of the continued AL use in Mali. The reduced prevalence of the 86**Y** allele in Mali, however, provides further support for the use of amodiaquine, a drug combined with sulfadoxine-pyrimethamine and used for seasonal malaria chemoprevention in children under five years in Mali.

The *Pfcr*t 76**T** mutation has also been shown to play a role in amodiaquine resistance in several studies in Africa [17]. Prior studies have demonstrated the selection of this allele after amodiaquine monotherapy [19] or ASAQ treatment [17, 20–22]. A lower prevalence of the *Pfcrt* 76**T** allele in the post-treatment was observed compared to pre-treatment isolates in this study, in contrast to previous reports. These unexpected findings can be explained by the fact that a majority (27) of the post-treatment isolates in our study were reinfection isolates and only four recrudescent isolates (among which three had the 76**T** mutation). The withdrawal of CQ for the treatment of malaria was followed by a steady decline of the prevalence of the *Pfcrt* 76**T** allele in many African countries [43]. However, this decline has varied by region, with West Africa still showing a steady prevalence of 58.3% [43]. The baseline prevalence of this allele in this study was 57.7%, similar to observations reported by others [41] who found equally high prevalence of this allele in Dangassa (64.3%) and Nioro-du-Sahel (42.5%). Whether this is a result of increased use of amodiaquine is unknown and additional surveillance studies are required.

Although investigation of molecular markers was performed on post-treatment samples (by combining samples with reinfections and recrudescent infection), association between observed parasite genotypes and recrudescent infection was not possible due to the small number of recrudescent infections (n = 9) which, when analysed alone, did not provide sufficient power. Nonetheless, monitoring of molecular markers of resistance to partner drugs during a therapeutic efficacy study is becoming increasingly feasible and provides a cost-effective tool for the early detection of decreases in parasite susceptibility to the drugs.

## Conclusion

This study demonstrated that day 28 PCR-corrected efficacy of both AL and ASAQ for uncomplicated malaria exceeded 90%, a WHO-recommended cut-off below which alternative treatments should be considered. Both uncorrected and PCR-corrected results suggested that ASAQ may be more efficacious, although the study was not designed to provide a comparison; however, this artemisinin-based combination is not employed routinely as a first-line malaria treatment because amodiaquine is paired with sulfadoxine-pyrimethamine and used throughout the country for seasonal malaria chemoprevention. Future studies in Mali should continue to monitor the efficacy of the current first-line ACT and consideration should also be given to evaluating artemisinin-based combinations, such as DP, with longer acting partner drugs.

## Supplementary Information

**Additional file 1: Table S1.** Kaplan Meier efficacy estimates and hazard ratios between AL and ASAQ, therapeutic efficacy monitoring, Mali, 2015–2016. **Table S2.** *msp1, msp2*, and *glurp* genotyping data (allele sizes are reported in base pairs (bp)).

## Data Availability

The datasets will be available on the WWARN (https://www.wwarn.org).
