# Supplementary material for: Therapeutic efficacy of artemether–lumefantrine and artesunate–amodiaquine for the treatment of uncomplicated Plasmodium falciparum malaria in Mali, 2015–2016
Source: Malar J. 2021 May 25;20:235. doi: 10.1186/s12936-021-03760-9 (PMC8146210; doi:10.1186/s12936-021-03760-9)
Supplement: Supplementary file 1 — Additional file 1: Table S1. Kaplan Meier efficacy estimates and hazard ratios between AL and ASAQ, therapeutic efficacy monitoring, Mali, 2015–2016. Table S2. msp1, msp2, and glurp genotyping data (allele sizes are reported in base pairs (bp)). [file 12936_2021_3760_MOESM1_ESM.doc]

Table S1: Kaplan Meier efficacy estimates and hazard ratios between AL and ASAQ, therapeutic efficacy monitoring, Mali, 2015–2016.

|  | **AL** | | **95% CI** | |  | **ASAQ** | | **95% CI** | | **Hazard Ratio** | **p-value** |
| --- | --- | --- | --- | --- | --- | --- | --- | --- | --- | --- | --- |
| **Uncorrected** |  | |  | |  |  | |  | |  |  |
| Kaplan Meier estimate – day 28 | 83.6% | | 78.8% | 88.6% |  | 93.2% | | 89.9% | 96.6% | 2.58 (1.4, 4.7) | 0.002^†^ |
| Kaplan Meier estimate – day 42 | 73.1% | | 67.4% | 79.3% |  | 84.4% | | 79.7% | 89.4% | 1.9 (1.2, 2.9) | 0.003^†^ |
| **PCR-corrected** |  |  |  |  |  |  |  |  |  |  |  |
| Kaplan Meier estimate – day 28 | 91.9% | | 88.3% | 95.5% |  | 97.3% | | 95.1% | 99.4% | 3.12 (1.2, 8.9) | 0.016^†^ |
| Kaplan Meier estimate – day 42 | 91.3% | | 87.6% | 95.1% |  | 96.8 | | 94.4 | 99.2 | 2.9 (1.2, 6.8) | 0.018^†^ |
| **Reinfections** |  | |  |  |  |  | |  |  |  |  |
| Kaplan Meier estimate – day 28 | 90.9% | | 86.9% | 95.0% |  | 95.7% | | 93.1% | 98.5% | 2.59 (1.42, 4.73) | 0.002^†^ |
| Kaplan Meier estimate – day 42 | 80.0% | | 74.6% | 85.8% |  | 87.2% | | 82.8% | 91.8% | 1.65 (1.0, 2.7) | 0.045^†^ |

AL: artemether lumefantrine

ASAQ: artesunate-amodiaquine

^†^p<0.05

Table S2: *msp1*, *msp2,* and *glurp* genotyping data (allele sizes are reported in base pairs (bp)).

|  | ***msp1* (bp)** | | | ***msp2* (bp)** | | ***glurp* (bp)** |  |  |  |
| --- | --- | --- | --- | --- | --- | --- | --- | --- | --- |
|  |  | | |  | |  |  |  |  |
| **Sample ID and day** | **K1** | **MAD20** | **RO33** | **FC27** | **IC1/3D7** | ***glurp* (bp)** | **Classification** | **Parasitemia** | **Treatment arm** |
| **SL009 D0** | 267 | 226 | 160 | 226 | 386 | 911 |  | 28625 | AL |
| **SL009 D14** | 259 | 219 | 160 | 297 | 481 | 632 | Reinfection | 23925 | AL |
| **SL010 D0** | 239;192 |  | 160 | 357 | 635;480 | 900 |  | 6125 | ASAQ |
| **SL010 D14** | 231 |  | 160 | 432 | 567 | 969 | Reinfection | 175 | ASAQ |
| **SL012 D0** | 207 |  |  | 381 | 478 | 1069 |  | 185875 | AL |
| **SL012 D35** |  |  | 160 | 429;373 | 676 |  | Reinfection | 125 | AL |
| **SL024 D0** |  |  | 160 | 570;223 | 576;511 | 614 |  | 12550 | ASAQ |
| **SL024 D42** | 161 | 210 |  | 347 | 669 | 611 | Reinfection | 3650 | ASAQ |
| **SL025 D0** | 180 | 222 | 160 | 373 | 549 |  |  | 2000 | ASAQ |
| **SL025 D35** | 280 |  |  | 380;320 | 469 |  | Reinfection | 125 | ASAQ |
| **SL027 D0** | 218 | 208 |  | 327 | 488 | 637 |  | 36250 | ASAQ |
| **SL027 D25** |  | 218 |  | 299 | 493 | NA | Recrudescence | 1050 | ASAQ |
| **SL030 D0** | 160 |  | 160 | 332 | 469 | 675 |  | 29975 | AL |
| **SL030 D19** | 154 |  | 160 | 337 | 476 | 670 | Recrudescence | 2005 | AL |
| **SL047 D0** | 236 | 211 | 0 | 312 | 529 | 734 |  | 6250 | ASAQ |
| **SL047 D24** | 0 | 204 | 0 | 323;343 | 410;418 | 0 | Reinfection | 28900 | ASAQ |
| **SL049 D0** | 261 |  |  |  | 615;517 | 745 |  | 26500 | AL |
| **SL049 D42** | 225 | 289 |  | 313 |  |  | Reinfection | 125 | AL |
| **SL052 D0** | 265;178 | 227;150 |  | 390;296;200 |  | 632 |  | 144400 | AL |
| **SL052 D25** | 260;170 | 230;143 |  | 397;292;202 |  | 623 | Recrudescence | 180300 | AL |
| **SL056 D0** |  | 210;130 |  | 389;292 | 564 | 614 |  | 2125 | ASAQ |
| **SL056 42** | 157 | 204 | 160 | 282 | 433 | 713 | Reinfection | 162825 | ASAQ |
| **SL057 D0** | 182 |  | 160 | 363;315;250 | 587;490 | 664;472 |  | 28750 | ASAQ |
| **SL057 D42** |  | 120 | 160 | 293;218 | 549;426 | 614 | Reinfection | 26050 | ASAQ |
| **SL059 D0** | 163 |  |  |  | 547 | 766 |  | 2250 | AL |
| **SL059 D25** |  | 200 |  | 359;311 | 472 | 584 | Reinfection | 1175 | AL |
| **SL060 D0** | 217 |  |  | 376;276 | 438 | 683 |  | 31000 | AL |
| **SL060 28** | 208 |  |  | 349;207 |  | 702;590 | Reinfection | 8050 | AL |
| **SL065 D0** |  | 229 |  | 322 | 557 | 581 |  | 2450 | ASAQ |
| **SL065 D35** |  | 130 |  | 362;325 | 535 |  | Reinfection | 11100 | ASAQ |
| **SL072 D0** | 250 |  | 160 |  | 651 | 664 |  | 2000 | AL |
| **SL072 D35** |  | 253 |  |  | 590 | 895 | Reinfection | 1400 | AL |
| **SL073 D0** |  | 276 |  |  |  |  |  | 2025 | AL |
| **SL073 D35** |  | 234 | 160 | 363;269 |  | 660 | Reinfection | 83350 | AL |
| **SL075 D0** | 270 | 221;230 |  | 313 | 461;390 | 603 |  | 62925 | AL |
| **SL075 D21** | 217 |  |  |  | 382 | 729 | Reinfection | 1050 | AL |
| **SL076 D0** | 253 |  |  | 262 | 380 | 657 |  | 18250 | AL |
| **SL076 D21** | 252 |  |  | 383;281 | 477 |  | Reinfection | 50 | AL |
| **SL078 D0** | 220 | 236 |  | 336 | 477 | 729 |  | 2125 | AL |
| **SL078 D21** |  | 220 | 160 | 372;269;181 | 464 |  | Reinfection | 25 | AL |
| **SL088 D0** | 290;249 |  |  | 361;285 | 418 | 709 |  | 2500 | ASAQ |
| **SL088 D35** | 210;221 |  |  | 311 |  |  | Reinfection | 31750 | ASAQ |
| **SL093 D0** | 260;227 |  |  | 315;205 | 504 | 713 |  | 97500 | AL |
| **SL093 D35** | 230;194 | 229 | 160 |  | 439 | 729 | Reinfection | 950 | AL |
| **SL094 D0** | 206 | 224 |  | 562;287 | 525;604;485 | 713 |  | 26625 | ASAQ |
| **SL094 D35** |  | 257 |  | 336 | 625;525 | 657 | Reinfection | 100 | ASAQ |
| **SL096 D0** | 238 | 211 |  | 302 | 685 | 906 |  | 78300 | ASAQ |
| **SL096 D35** | 250;211 |  |  | 518;287 | 665;544 | 719;406 | Reinfection | 2675 | ASAQ |
| **SL098 D0** | 247;146 |  | 160 | 409;305;204 | 707;609 | 510 |  | 4400 | ASAQ |
| **SL098 D42** | 181 | 202 |  | 532 | 328 | 760 | Reinfection | 21750 | ASAQ |
| **SL099 D0** | 230;288 |  |  | 302 | 613;456 | 517 |  | 19875 | ASAQ |
| **SL099 D42** | 220 |  |  | 334;220 | 740 |  | Reinfection | 1250 | ASAQ |
| **SL105 D0** | 250 | 245 |  | 575;341 | 597;534 | 1045 |  | 43875 | AL |
| **SL105 D35** | 216 | 190 |  | 469 | 561;388 |  | Reinfection | 750 | AL |
| **SL108 D0** | 185 |  |  | 557 | 640;325 | 596 |  | 6100 | ASAQ |
| **SL108 D35** | 176 |  |  | 520;482 | 503;583 | 634 | Reinfection | 22750 | ASAQ |
| **SL110 D0** | 187 | 187 | 160 | 536;307 | 700;633;504 | 828 |  | 8000 | AL |
| **SL110 D17** | 267;214 |  |  | 431 | 435;312 |  | Reinfection | 200 | AL |
| **SL111 D0** | 183 |  |  | 564 |  | 880 |  | 75000 | ASAQ |
| **SL111 D26** | 213 |  |  |  | 584 | 860 | Reinfection | 25000 | ASAQ |
| **SL117 D0** | 277;205;124 |  |  | 332 |  | NA |  | 97500 | ASAQ |
| **SL117 D14** |  | 217 |  |  | 556 | NA | Reinfection | 7500 | ASAQ |
| **SL121 D0** | 227 | 238 | 160 | 394;338;289 | 548 | 645 |  | 26950 | ASAQ |
| **SL121 D28** | 230 | 225 |  | 300;225 | 517;433 |  | Reinfection | 550 | ASAQ |
| **SL128 D0** | 287;222 | 204 |  | 381;296 | 575;473;369 | 626 |  | 33650 | AL |
| **SL128 D21** |  | 227 | 160 | 376;286 | 500;406 |  | Reinfection | 750 | AL |
| **SL130 D0** | 246;184 |  |  | 281 | 483;392 | 593 |  | 34500 | ASAQ |
| **SL130 D35** | 204 |  |  | 346;289 | 538;439 | 630 | Reinfection | 75 | ASAQ |
| **SL139 D0** | 300;255 |  | 160 | 300 | 495;394 | 627 |  | 73150 | AL |
| **SL139 D35** | 200;197 |  | 160 | 393;299 | 500;373 | 837 | Reinfection | 1750 | AL |
| **SL143 D0** |  | 232 | 160 | 432 |  | 843 |  | 5625 | AL |
| **SL143 D35** | 180 |  |  |  | 500 | 884 | Reinfection | 2425 | AL |
| **SL148 D0** | 208 |  | 160 | 356 | 645 | 753 |  | 5175 | ASAQ |
| **SL148 D42** | 255 |  |  | 436 | 537 | 754 | Reinfection | 4740 | ASAQ |
| **SL156 D0** | 296;140 | 219 |  | 275;220 | 475 | 670 |  | 9175 | AL |
| **SL156 D20** |  | 250 |  | 306;260 | 634;533 | 549 | Reinfection | 23750 | AL |
| **SL160 D0** | 261;229 | 238;212 |  | 408 | 544 | 699;544 |  | 56600 | ASAQ |
| **SL160 D28** | 202 |  |  | 307 | 507;441 | 0 | Reinfection | 175 | ASAQ |
| **SL163 D0** |  | 227 |  |  | 595 | 676 |  | 7250 | AL |
| **SL163 D27** | 180 |  | 160 | 435 |  | 745 | Reinfection | 81300 | AL |
| **SL164 D0** | 163 | 247 | 160 | 377;303 | 500;434 | 696 |  | 4075 | AL |
| **SL164 D40** | 170 | 247 | 160 | 372;310 | 500 | 702 | Recrudescence | 2575 | AL |
| **SL168 D0** | 239 |  |  | 398;304 | 469 | 462 |  | 13500 | AL |
| **SL168 D21** |  | 244 |  | 296 | 454;398;331 | 585 | Reinfection | 25 | AL |
| **SL176 D0** | 224 |  |  | 301 | 514 | 630 |  | 25000 | AL |
| **SL176 D35** | 226 |  |  | 406;328;288 | 525 | 643 | Reinfection | 6250 | AL |
| **SL185 D0** | 214 |  |  | 392;288 | 431;347 | 679 |  | 3025 | AL |
| **SL185 D42** | 227 |  | 160 | 281 | 468 | 743 | Reinfection | 25 | AL |
| **SL189 D0** | 200 | 225 |  | 333 | 510 | 704 |  | 23750 | ASAQ |
| **SL189 D40** | 193 | 218 |  | 339 | 500 | 696 | Recrudescence | 525 | ASAQ |
| **SL192 D0** |  |  | 160 |  | 595 | 720 |  | 18500 | AL |
| **SL192 D35** |  | 203 | 160 |  | 633 | 645 | Reinfection | 125 | AL |
| **SL195 D0** | 159 |  | 160 | 468 | 504 | 689 |  | 6250 | ASAQ |
| **SL195 D21** | 164 |  | 160 | 460 | 510 | 696 | Recrudescence | 5800 | ASAQ |
| **SL205 D0** | 240 |  |  | 462;344;300 | 619;513 | 669;619 |  | 9350 | AL |
| **SL205 D28** | 255 |  |  | 356 | 700;594 | 709 | Reinfection | 33500 | AL |
| **SL214 D0** | 185 |  |  | 366 | 576 | 667 |  | 3050 | AL |
| **SL214 D28** | 250 | 247 |  | 356 | 679 | 659 | Reinfection | 4125 | AL |
| **SL216 D0** | 245 |  |  | 361;323 |  | 768 |  | 8425 | AL |
| **SL216 D35** | 189 |  |  | 310 |  | 854 | Reinfection | 11125 | AL |
| **SL221 D0** |  | 140 |  | 351;263 |  | 770 |  | 12775 | AL |
| **SL221 D35** | 223 |  |  | 347;223 | 590 | 874 | Reinfection | 8825 | AL |
| **SL236 D0** | 213 | 251 | 160 | 350 | 550 | 756 |  | 3625 | ASAQ |
| **SL236 D21** | 195 | 242 | 160 | 344;263 | 550 | 764 | Recrudescence | 1050 | ASAQ |
| **SL238 D0** | 140 |  |  | 346;308 | 550 | 600 |  | 2975 | AL |
| **SL238 D35** | 157 | 130 |  | 406;349;303 | 609;490 | 511 | Reinfection | 15900 | AL |
| **SL239 D0** | 140 |  |  | 341 | 539 | 724 |  | 15650 | AL |
| **SL239 D35** |  | 140 |  | 333 | 516 | 768;704 | Reinfection | 75 | AL |
| **SL246 D0** | 140 |  |  | 387;339 | 534 | 650 |  | 4500 | AL |
| **SL246 D28** | 224 |  |  | 330 | 629;418 | 780;660 | Reinfection | 1950 | AL |
| **SL247 D0** |  | 160 |  | 327;256 | 509 | 681 |  | 8850 | ASAQ |
| **SL247 D35** | 221 |  |  | 386;296 | 500 | 774 | Reinfection | 2600 | ASAQ |
| **SL250 D0** | 228 |  |  | 357;317 | 387 | 604 |  | 11250 | ASAQ |
| **SL250 D21** | 200 |  | 160 | 330 | 383;336;299 | 609;556 | Reinfection | 25 | ASAQ |
| **SL253 D0** | 232 | 211 |  | 592;548 | *306;224* | 675 |  | 8175 | AL |
| **SL253 D32** | 213 |  | 160 |  | 334 | 574 | Reinfection | 36350 | AL |
| **SL260 D0** |  |  | 160 | 475 | 339 | 439 |  | 14100 | AL |
| **SL260 D28** |  | 222 | 160 | 374;310;268 | 567;421;377 |  | Reinfection | 500 | AL |
| **SL263 D0** | 218 | 212 |  | 335 | 530 | 753 |  | 95625 | AL |
| **SL263 D21** | 203 | 193 |  | 370 | 648 | 834 | Reinfection | 50 | AL |
| **SL269 D0** | 149 | 187 |  | 383 | 507 | 656 |  | 3750 | AL |
| **SL269 D42** | 142 |  |  | 343 | 539 | 653 | Reinfection | 26000 | AL |
| **SL270 D0** |  | 168 | 160 | 300;338 |  | 637 |  | 46475 | AL |
| **SL270 D21** | 127 |  | 156 | 330 | 400;530 | NA | Recrudescence | 50 | AL |
| **SL280 D0** | 146 |  |  | 256 | 494 | 773 |  | 66900 | ASAQ |
| **SL280 D32** | 192 |  |  | 282 | 500 | 780;671 | Reinfection | 13450 | ASAQ |
| **SL282 D0** | 215 | 196 |  | 304 | 461 | 890 |  | 23000 | ASAQ |
| **SL282 D35** |  | 200 |  | 220 | 381 | 656 | Reinfection | 425 | ASAQ |
| **SL286 D0** | 196 | 255 | 160 | 305 | 447 | 707 |  | 34050 | ASAQ |
| **SL286 D35** | 199 | 257 |  | 300 | 489 | 681 | Reinfection | 29250 | ASAQ |
| **SL288 D0** |  | 290 |  |  | 583;471 | 732 |  | 52275 | ASAQ |
| **SL288 D28** | 143 |  | 160 | 400 | 650;568 | 623 | Reinfection | 100 | ASAQ |
| **SL291 D0** | 149 | 260 | 160 | 329 | 538 | 684 |  | 19800 | AL |
| **SL291 D27** | 152 | 254 | 160 | 338 | 538 | 692 | Recrudescence | 16000 | AL |
| **SL297 D0** | 235;174 | 206 | 160 | 385 | 558 | 748 |  | 5350 | ASAQ |
| **SL297 D35** | 223 |  |  | 395;308 | 662 | 872 | Reinfection | 200 | ASAQ |
| **SL311 D0** |  | 257 | 160 | 553;277 | 563;659 | 604 |  | 44125 | ASAQ |
| **SL311 D28** |  | 231 |  |  |  | 765 | Reinfection | 25 | ASAQ |
| **SL377 D0** | 213 | 224 | 160 | 313 |  | 835 |  | 19300 | AL |
| **SL377 D35** | 245 | 249 |  | 302 |  | 746 | Reinfection | 1625 | AL |
| **SL381 D0** |  | 290 | 160 | 538;386;295 | 300 | 778 |  | 6000 | AL |
| **SL381 D21** |  | 275 |  | 290 |  | 730 | Reinfection | 22500 | AL |
| **SL384 D0** |  | 284 |  | 475;375;259 | 698;513 |  |  | 3075 | AL |
| **SL384 D35** |  | 243 |  | 521 |  |  | Reinfection | 875 | AL |
| **SL393 D0** | 249 | 257 |  | 498;400;285 | 709;332 | 648 |  | 42250 | AL |
| **SL393 D42** |  | 193 |  | 351 | 473;376 | 714 | Reinfection | 78000 | AL |
| **SL430 D0** |  | 280 | 160 | 317 |  |  |  | 32000 | AL |
| **SL430 D42** |  | 276 |  | 405;403 |  | 714 | Reinfection | 20000 | AL |
| **SL448 D0** |  | 240 |  | 427;263 | 600 | 871 |  | 10850 | AL |
| **SL448 D21** |  | 287 |  | 317 | 612 |  | Reinfection | 5875 | AL |

Band sizes were scored using UVP automated Gel Image system and compared across the three markers. If there was at least one matching band in any allelic family for all three markers, the recurrence was classified as a recrudescence (regardless of whether there were additional or missing alleles). If there were no shared alleles for at least one marker, the recurrence was classified as a reinfection. If there were not amplification products resulting in sharp, defined bands in both the pre-treatment and day of recurrence samples for a gene, that gene was not used to distinguish between recrudescence and reinfection, but the aforementioned classification criteria were applied for the genes that were amplified.
